# Recent Shifts in Global Governance: Implications for the Response to Non-communicable Diseases

**DOI:** 10.1371/journal.pmed.1001487

**Published:** 2013-07-23

**Authors:** Devi Sridhar, Claire E. Brolan, Shireen Durrani, Jennifer Edge, Lawrence O. Gostin, Peter Hill, Martin McKee

**Affiliations:** 1 School of Social and Political Sciences, University of Edinburgh, Edinburgh, United Kingdom; 2 Blavatnik School of Government, University of Oxford, Oxford, United Kingdom; 3 School of Population Health, University of Queensland, Brisbane, Australia; 4 O'Neill Institute for National and Global Health Law, Georgetown University, Washington, D.C., United States of America; 5 European Observatory on Health Systems and Policies, London School of Hygiene and Tropical Medicine, London, United Kingdom

## Abstract

Devi Sridhar and colleagues discuss how three major trends in global governance, the rise of emerging economies, the increase in multi-bi financing and institutional proliferation, have ramifications for whether NCDs will be included in the post-2015 Sustainable Development Goals agenda.

*Please see later in the article for the Editors' Summary*

**Summary Points**

- Despite evidence of links between non-communicable diseases (NCDs) and development, these diseases and their risk factors were not included in the Millennium Development Goals (MDGs).
- Three major trends in global governance—the rise of emerging economies, the increase in multi-bi financing, and institutional proliferation—have implications for whether NCDs will be included in the post-2015 Sustainable Development Goals (SDGs) agenda.
- While emerging economies are influential in global governance, it is not clear that the interests of poorer countries—or even health—will be advanced.
- If NCDs are included in the new health goals, it likely will be via the broad umbrella of healthy life expectancy (HLE), or the sector-specific target of universal health coverage (UHC) or access.
- UHC or HLE as currently conceived are unlikely to adequately incorporate NCDs that require alternative health system mechanisms and clear governmental intervention.

## Introduction

As the 2010 Global Burden of Disease study confirmed, non-communicable diseases (NCDs) (primarily cardiovascular disease, cancer, chronic respiratory disease, and diabetes) are now the major cause of death and disability across the world [1]. In 1990, 47% of disability-adjusted life years worldwide were attributable to communicable, maternal, neonatal, and nutritional deficits, 43% to NCDs, and 10% to injuries. By 2010, this had shifted to 35%, 54%, and 11%, respectively. Over 80% of NCD-related deaths occur in low- and middle-income countries, with lower socio-economic groups the worst affected in terms of morbidity, mortality, and loss of economic opportunity [2]. These figures do not account for the health and economic burdens of the wide range and prevalence of mental health conditions, which are seen by many as leading NCDs.

The increasing importance of NCDs has several implications for development. First, unlike most acute infectious diseases, the often chronic and debilitating course of NCDs impedes social and economic development, deepening inequalities, and initiates a cycle of disability and health costs-related poverty [3],[4]. Second, as most NCDs share common major risk factors and present similar challenges for clinical management, an integrated response is required, avoiding the health care “silos” that have arisen as a consequence of the narrow focus on HIV, malaria, and tuberculosis (TB) by international donors: well-funded projects that operate in isolation from national health systems may fail to address wider health care needs [5],[6].

Fifteen years ago, in the wake of rising concerns over the lack of progress in reducing global poverty, all 189 UN Member States committed themselves to eight goals aimed at reducing poverty [7]. Yet despite the evidence of a strong association between NCDs and development, these diseases and their shared risk factors were not included in the Millennium Development Goals (MDGs) [8].

As the 2015 deadline for achieving the MDGs approaches, a new development agenda is being mapped out to advance the progress made towards the MDGs while addressing remaining gaps and meeting the complex political and economic governance challenges of the post-2015 landscape. Will the new sustainable development goals (SDGs) be able to respond effectively to the rising tide of NCDs? In this paper we examine three major trends in global governance and their implications for post-2015 progress relating to NCDs.

## Trend 1: Rise of the Emerging Economies

As we move into the second decade of the 21st century, global power is shifting yet again, two decades after the changes that followed the collapse of the USSR. The power of a few rich countries (notably the USA, United Kingdom, Germany, and France) to shape the global agenda is being challenged by the growing economic power, of what have been termed the BRICS countries (Brazil, Russia, India, China, and South Africa) and the so-called CIVETs (Colombia, Indonesia, Vietnam, Egypt, and Turkey).

What does this mean for NCDs? On the one hand, NCDs are rising on the agendas of established international bodies. In 2011 the United Nations held a High Level Meeting on NCDs, only the second time that it had elevated health to this level [9]. The first such meeting was on HIV/AIDS, following which the G8 took up the torch, creating new institutions such as the Global Fund to Fight AIDS, Tuberculosis and Malaria, and prioritizing certain issues such as universal access to anti-retrovirals. Yet it is not clear whether the High Level Meeting on NCDs, which was driven by a group of small countries, many in the Caribbean, will lead to similarly sustained action. Moreover, the G8 have not considered global health in either their 2011 or 2012 agendas [10] although the 2010 G8 meeting did result in the Muskoka Accord for maternal health. To the extent that health will feature on the G8's 2013 agenda, it is likely to be in the context of liberalization of trade in health services. Although the locus of global decision-making is considered to be shifting from the G8 to the G20, it is not clear that the latter body is willing to assume even the limited interest in global health governance seen with the G8 [11], despite hopes from the health community that it would do so [12]. Moreover, it is not apparent from their previous engagement in global health debates that newer G20 states have a major interest in NCDs. Thus, having more emerging economies at the table does not necessarily mean clearer articulation of an effective NCD response.

Health has also been largely absent from the agendas of the BRICS countries, now forming a semi-official grouping. While they are increasingly influential in finance and trade, they have had only limited influence thus far in global health. The fact that the relatively economically stable BRICS have not stepped up their commitments to the Global Fund, the GAVI Alliance, or WHO has raised questions about their commitment to global health leadership in the long term (Table 1) [13]–[19]. Domestically, Russia and to a lesser extent China, are the only two BRICS countries as of yet to address NCDs in a substantive manner.

**Table 1:** BRIC financial contribution to key global health institutions and amount received from Global Fund and GAVI [14]–[19].

Country	Global Fund Contributions (Cumulative to End 2012, $US Millions)	Global Fund Amount Received (Cumulative to Date, $US Millions)	GAVI Contributions (Cumulative to 2012, $US Millions)	GAVI Amount Received (Cumulative to 2012, $US Millions)	WHO Core Contributions (2012, $US Millions)	WHO Extrabudgetary Contributions ($US Millions)
Brazil	0.0	39.1	0.0	0.0	7.5	0.03
Russia	297.0	372.0	24.0	0.0	7.4	6.1
India	10.0	1,019.9	0.0	94.0	2.5	0.015
China	25.0	763.3	0.0	38.7	14.8	0.4
S. Africa	10.3	350.6	6.0	0.0	1.8	0.0

There is some evidence of global health achieving a higher priority elsewhere, exemplified by the Foreign Policy and Global Health Initiative, which draws its leadership largely from the South and consists of five Southern (Brazil, Indonesia, Mexico, Senegal, and Thailand) and two Northern (France and Norway) countries. Yet, with the exception of the Caribbean Community (CARICOM) [20], it is uncertain whether the rise of newly emerging economies will mean global engagement with NCDs.

In summary, although emerging economies are clearly influential in global governance, there is little evidence of commitment to the NCD agenda, and it does not follow that they will advance the interests of poorer countries—or even health. To the extent that these countries do engage in health, it has been issue-specific, such as on access to essential medicines, technological cooperation, or on Trade-Related Aspects of Intellectual Property Rights (TRIPs)—all areas where health is incidental to trade concerns. Furthermore, global health engagement by emerging powers is often driven by regional concerns, which explains the re-invigoration and creation of regional bodies in health, particularly in Latin America. In this changing environment there is a danger of health slipping off the agenda of the traditional economic and political powers but not being taken up by the emerging ones.

## Trend 2: Rise (and Fall) of Multi-bi Financing

Over the past decade, most of the growth in multilateral funding has been through the channel of “multi-bi aid” [21]. This refers to the practice of donors choosing to route non-core funding, earmarked for specific sectors, themes, countries, or regions, through multilateral agencies. At first glance the funding looks multilateral, but upon closer inspection, it is essentially bilateral. Examples of multi-bi aid include voluntary contributions within the WHO, trust funds within World Bank, the Global Fund, and the GAVI Alliance. Since 2002, global health donors have increasingly prioritized multi-bi aid at the expense of more traditional forms of multilateral aid as a proportion of all development assistance for health [22]. Multi-bi aid increased as a proportion of all aid at a rate of approximately 1.5–2.0 percentage points per year over this time period [22].

The rise of multi-bi aid has three implications for the NCD agenda. First, an analysis of the WHO's expenditures shows a significant misalignment with the burden of disease, both globally and at regional levels, with the additional voluntary resources least well aligned [23]. In 2008–2009 of the WHO's regular budget, 25% of funds were allocated to infectious disease, 8% for NCDs, and roughly 4.7% for injuries, which when compared with the global distribution of DALYs noted above indicates a disproportionate share of resources going to the first of these at the expense of the remaining two. However, the WHO's extra-budgetary funding for 2008–2009 is even further out of alignment, with 60% allocated primarily to infectious diseases, while only 3.9% was used for NCDs and 3.4% for injuries.

Second, the emergence of new multi-stakeholder global health funding institutions such as the Global Fund and the GAVI Alliance has signaled a major shift in global cooperation, one in which voting rights and board membership is granted to the private sector and philanthropic organizations and legitimacy is claimed through improving specific measurable health outcomes [21]. Given the aggressive tactics the tobacco, alcohol, and food industries have used to oppose regulation addressing key NCD risk factors, it will be difficult to have industry at the table while addressing the root causes of the pandemic [24].

Third, an analysis by Grepin and Sridhar shows that the movement towards multi-bi aid reversed since the onset of the global financial crises with donors decreasing their contributions to the GAVI Alliance, the Global Fund, and UNAIDS since 2008 [22]. This is particularly true of the ten largest global health donors, where this channel of funding decreased by nearly 6% of all development assistance for health during 2008–2009. Thus, it is unlikely that in the current financial climate new funds will be available to address NCDs. Given this situation, national governments will have to bear almost all the costs of responding to NCDs and will have little external incentive to prioritize these diseases [25].

## Trend 3: Institutional Proliferation

Since 2000, more and more global health institutions and initiatives have been created such as the GAVI Alliance, the Global Fund, and the Global Alliance for Improved Nutrition. However, they remain largely uncoordinated, focused on vertical disease-specific programs, and lack rigorous assessment [26],[27]. Initiatives designed to support coherence among global players such as the International Health Partnership (IHP+) and Health 8 (H8) have remained largely focused on vertical global health program delivery rather than taking a role in leading governance for health as a global public good [28].

This institutional proliferation has two implications for the future NCD agenda. First, only a handful of these actors are interested in or focusing on the drivers of the NCD epidemic. According to the Institute for Health Metrics and Evaluation, only US$185 million of the US$28.2 billion spent globally on development assistance for health in 2010 was dedicated to NCDs [29]. Donors spent US$300 for each year lost to disability from HIV/AIDS, US$200 for malaria, and US$100 for TB, but less than US$1 for NCDs. Nearly half of the development assistance for NCDs in 2010 derived from a single source: the Bloomberg Family Foundation [30].

Second, the drivers of NCDs are intricately connected with the policies of non-health sectors [30]. Multi-sector participation has already begun on health issues at the state-level through inter-ministerial working groups focused on global health, the reduction of health inequities, and HIV/AIDS prevention in Australia, Canada, India, Norway, Sweden, Switzerland, Thailand, Uganda, United Kingdom, and United States [31]–[33]. Institutional incentive structures to engage other sectors in negotiations about health are crucial to raising the profile of health-related priorities in other policy communities at the all levels of governance. However, the WHO and other global health agencies presently lack the resources and mechanisms to meaningfully participate in policy issues like trade, agriculture, security, and climate change [29].

## Post-2015: Universal Health Coverage and Healthy Life Expectancy

If NCDs are to be included in the new health goals, it seems most likely to be through the the sector-specific target of universal health coverage (UHC) or access or the broad umbrella of healthy life expectancy. UHC has received particular prominence recently (Box 1). In January 2012, the Bangkok Statement on UHC committed to “raise universal health coverage on the national, regional and global agendas, and to advocate the importance of integrating it into forthcoming United Nations and other high-level meetings related to health or social development” [34]. In April 2012 the Mexico City Political Declaration on UHC emphasized universal coverage as “an essential component of sustainable development” and its inclusion “an important element in the international development agenda” [35]. In June 2012 the Rio+20 resolution explicitly recognized UHC, seeking “to strengthen health systems towards the provision of equitable universal coverage” [36]. Later in 2012, a WHO Discussion Paper on the Post 2015 health agenda, identified UHC as a “way of bringing all programmatic interests under an inclusive umbrella” [37]. On 12 December 2012 UHC received unequivocal endorsement from the UN General Assembly (including the United States) in approving a resolution on UHC, confirming the “intrinsic role of health in achieving international sustainable development goals” [38].

**Box 1. Political History of UHC**

**Date Event**

1975 Health for All. (WHO)

1978 Declaration of Primary Health Care, Alma Ata. (WHO/UNICEF)

1987 Bamako Initiative on Health Financing. (UNICEF/WHO)

1993 World Development Report 1993: Investing in Health. (World Bank)

2000 World Health Report 2000. Health systems: Improving performance. (WHO)

2000 UN Millennium Declaration

2001 Adoption of Millennium Development Goals

2002 Commission on Macro-Economics and Health. (WHO)

2005 Paris Declaration on Aid Effectiveness. (OECD-DAC)

2005 GAVI Alliance Health Systems Funding Window. (GAVI)

2006 Global Fund to fight AIDS, TB and Malaria Health Systems Funding Window. (Global Fund)

2007 International Health Partnerships Plus (IHP+)

2007 Everybody's Business. Strengthening Health Systems to Improve Health Outcomes. WHO's Framework for Action. (WHO)

2007 Inaugural meeting of H8 (Health 8)

2008 World Health Report 2008. Primary Health Care: Now more than ever. (WHO)

2008 G8 Commitment to strengthening Health Systems, Toyako.

2009 High Level Taskforce on Innovative Financing for Health Systems

2009 Health Systems Funding Platform. (Global Fund/GAVI/World Bank/WHO)

2010 World Health Report 2010. Health systems financing: The path to universal coverage. (WHO)

2010 1st Global Symposium on Health Systems Research, Montreux.

2011 Universal Health Coverage, WHO General Assembly

2012 Bangkok Statement on Universal Health Coverage, Prince Mahidol Award Conference

2012 Mexico International Forum on Universal Health Coverage. (WHO)

2012 2nd Global Symposium on Health Systems Research, Beijing.

2012 UN General Assembly: Universal Health Coverage declared a UN global goal.

2013 World Health Report 2013. Research for universal health coverage. (WHO)

As the above developments indicate, the post-2015 health discussions have been centered on UHC and its link to WHO's revitalization of Primary Health Care. In our opinion, this enthusiasm has been tempered by confusion as to what UHC actually is, as well as the fear of failure from previous attempts such as “Health for All.” For example, in 2005 the World Health Assembly officially defined the achievement of UHC as “access to key promotive, preventive, curative and rehabilitative health interventions for all at an affordable cost, thereby achieving equity in access” [39]. Yet, elsewhere, UHC has been construed as national service delivery, national service coverage, financial protection, and national health insurance and related reforms. We believe that it is unclear what health services UHC covers (e.g., whether it fully covers public health services such as sanitation, vector abatement, and tobacco control), and questions arise over whether UHC includes only services within a state's health sector or services and interventions outside the health sector [40],[41].

If UHC is to become a new development goal, we argue that baselines for achievement of UHC must be agreed and developed in post-MDG negotiations and adapted to country circumstance, fiscal realities, and community priority. Currently it is not clear that policy-makers have considered how to integrate the response to NCDs with the scaling up of basic care through strengthening primary health care. For example, the USSR was successful in scaling up measures against infectious diseases, but failed to tackle the key drivers of NCDs [42]. Most importantly, we feel that UHC will have limited impact on the rising tide of NCDs without targets and funding to reduce risk factors—requiring a prevention, public health, and “all of society” approach.

Recent discussions in Botswana highlight a move towards the broad umbrella of healthy life expectancy (HLE), or “maximizing healthy lives,” measured as “reducing healthy years of life lost” [43]. But will HLE result in a better response to NCDs? Civil society organizations have started to champion HLE as the best vehicle to address the social determinants of health, thus creating space to address the root causes of the NCD crisis. On the other hand, HLE could also be used to push for individual responsibility for health and reframing unhealthy behaviors as personal choices, ignoring the circumstances within which those choices are made. HLE also makes it harder to tie health outcomes to state or institutional responsibility. Thus, could NCDs be addressed in such health goals? At face value, yes. But it is not clear in our view whether UHC or HLE as currently conceived will adequately incorporate the prevention and treatment of NCDs, which require alternative health system mechanisms and clear responsibility placed on the state for ensuring a healthy environment.

## Conclusion

In the post-2015 debate, almost no attention has been given to the global governance structures necessary to support the attainment of the new goals. It is generally agreed that we need 21st-century innovative structures that go beyond the WHO “command and control” model, but little detail is given on institutional responsibility, monitoring, and evaluation and accountability [44]. For NCDs in particular, the global response requires more than new funding or financing mechanisms. It requires global regulation of the key vectors of the epidemic, as well as linkages between health and the other areas discussed as part of the SDGs agenda such as agriculture and food security, environment, trade, urban development, energy policies, education, poverty alleviation, and gender equity. This necessity points to both the key role of the WHO as well as the inherent limitation in making the agency the focal point for the response. The WHO is the only global health body with the power to create international law, and given its success in legislating against tobacco (The Framework Convention on Tobacco Control), similar “hard law” mechanisms for other main drivers of the NCD epidemic such as alcohol and processed food are certainly feasible [45]. At the same time, for multi-sectoral convergence to become a reality, various agencies of the UN must act in concert to catalyze, support, and monitor such collaboration [9].

While the epidemiological evidence is clear on the rising burden of NCDs across geographic boundaries, the current post-2015 discussions and larger global governance trends create challenges to addressing this burden effectively. These political and economic influences need to be considered carefully if NCDs are not to be left behind again.
